# The Modern "Hospital"

**Published:** 1903-06-13

**Authors:** 


### The Hospital.

A Journal of

The Medical Sciences and Hospital Administration.

Vol. XXXIV.—No. 872. Edited by Sir Henry Burdett, K.C.B. [June 13, 1903.

### The Modern "Hospital."

Sir Frederick Treves made a most forcible speech on behalf of the hospitals at the Mansion House on Monday, which will be found in another column. That speech carried conviction with it, because Sir Frederick Treves spoke the truth out of fulness of knowledge, the result of great experience in hospital work. We welcome Sir Frederick Treves, who by this speech has worthily taken up his position as a leader of the medical profession. The modern hospital is wonderful in its results; but the marvel of it is dependent upon the fact that the whole of the progress in science, represented by the methods of treatment in force to-day, owes its existence to the voluntary service of a few zealous men who, at their own cost, have made the discoveries by which alone it has been possible to attain the results achieved. Thirty years ago the mortality in our hospitals after major surgical operations was roughly 33 per cent.; in other words, one out of every three patients who underwent a severe operation died. To-day the mortality from severe operations is about 3 per cent.—that is to say, that there are only three fatal cases on an average out of every hundred serious operations performed. Again the average stay of a patient in some of the great hospitals of London was formerly 35 days, but that average has now been reduced to 19 days, thanks to the improvements referred to. This diminution in the days of hospital residence represents to the artisan classes of London every year in the aggregate something like one million working days. It is, therefore, but the truth to state that the actual dividend in results earned by the hospitals under modern methods of treatment represents to the residents in every large centre of population a very considerable sum in pounds sterling every year.

The late Sir Charles Trevelyan rightly called public attention to the abuse of hospitals in the material sense, when it was shown by statistics that the number of patients treated at the London hospitals was as one to four of the total population. To-day over two million patients are treated in the hospitals and medical institutions of London every year, so that the proportion of hospital patients to the population has increased from one in four to nearly one out of every two residents in London. There can be no doubt, therefore, when these figures are considered, that an increasing number of people receive treatment at the hospitals who can well afford to pay a portion or the whole of its cost. Their excuse is, as Sir Frederick Treves well pointed out, that all Londoners who may be afflicted with the more serious forms of disease and accident, with the exception of the very wealthy, require the facilities for treatment which they can only obtain in the modern hospital. Put thus concretely, the facts demand the serious attention of all who are responsible for the administration of our hospitals and also of the public at large. If the present state of affairs is to continue unchanged, and without modification, the time will soon arrive when the modern hospitals supported by voluntary contributions will number among their patients the majority of those who are seriously ill, whatever their social position may be. It follows that the whole system of medical relief in this country is ripe for reconsideration. The actual facts must be recognised and provided for by a system, which will afford the fullest opportunities to every patient to pay according to his means for the hospital care and treatment which he may require.

We have dealt so far with the modern hospital from the point of view of hospital supporters and hospital patients. There remains the still greater question of the duty and moral obligation placed upon every thinking man and woman, irrespective of their social position and means, to recognise the duty of contributing to the support of the hospitals in the days of their health. Quoting Sir Frederick Treves once more, it must not be forgotten that the old name for hospital was the guest-house. That is to say, that every sick man or woman who enters a hospital supported by the free gifts of the wealthy and well-to-do amongst us becomes the guest of the man of means in the days of his or her necessity. To state this is to enforce once more "that the real test of the greatness of a man is the strength of his compassion. The one movement which lifts a man up to true nobility is the movement which brings him down to the needs and sufferings of his brethren." So the modern hospital, supported by voluntary contributions, constitutes one of the noblest possessions of the British people. The modern hospital could never have attained to the efficiency at which it has arrived, nor could it have achieved the results we have here recorded in its battle with disease and death, had it not been for the noble generosity of those, who have proved the greatness of their manhood by the strength of their compassion for the wayfaring man struck down by disease or accident. We do not believe it is possible that the day will ever come when England will consent to the removal from the walls of our hospitals of the noble words "Supported by Voluntary Contributions."

Sir Frederick Treves showed, by a striking example, the depth of the gratitude of the individual sufferer, who finds relief, as the guest of the generous citizen, in one of our hospitals. The influence which is exercised by the principles upon which these institutions are supported in England to day, based as it is upon love, and owing most of its force to the strength of the compassion of the more thoughtful for the least fortunate of our race, renders the modern hospital a tower of strength. It constitutes a bond of union, which knits all classes together and humanises the whole nation. During the last 20 years a few earnest men, recognising the momentous importance of continuing the voluntary system of hospital support, have continuously laboured to place the finances of the hospitals upon a sound and permanent basis. That end was never nearer of attainment than it is to-day. We believe, indeed, that the support which the King and Queen Alexandra, with the members of the Royal house, have given to Hospital Sunday this year, will accomplish all that is necessary to supply ample funds, through voluntary contributions, for every purpose of the modern hospital.

